# Whole-genome sequence of an evolved *Clostridium pasteurianum* strain reveals Spo0A deficiency responsible for increased butanol production and superior growth

**DOI:** 10.1186/s13068-015-0408-7

**Published:** 2015-12-24

**Authors:** Nicholas R. Sandoval, Keerthi P. Venkataramanan, Theodore S. Groth, Eleftherios T. Papoutsakis

**Affiliations:** Department of Chemical and Biomolecular Engineering and the Delaware Biotechnology Institute, University of Delaware, 15 Innovation Way, Newark, DE 19711 USA; Department of Biological Sciences, University of Delaware, Newark, USA

**Keywords:** Butanol, *Clostridium pasteurianum*, Spo0A, Methylome, Mutagenesis, SMRT sequencing

## Abstract

**Background:**

Biodiesel production results in crude glycerol waste from the transesterification of fatty acids (10 % w/w). The solventogenic *Clostridium pasteurianum*, an anaerobic Firmicute, can produce butanol from glycerol as the sole carbon source. Coupling butanol fermentation with biodiesel production can improve the overall economic viability of biofuels. However, crude glycerol contains growth-inhibiting byproducts which reduce feedstock consumption and solvent production.

**Results:**

To obtain a strain with improved characteristics, a random mutagenesis and directed evolution selection technique was used. A wild-type *C. pasteurianum* (ATCC 6013) culture was chemically mutagenized, and the resulting population underwent 10 days of selection in increasing concentrations of crude glycerol (80–150 g/L). The best-performing mutant (M150B) showed a 91 % increase in butanol production in 100 g/L crude glycerol compared to the wild-type strain, as well as increased growth rate, a higher final optical density, and less production of the side product PDO (1,3-propanediol). Wild-type and M150B strains were sequenced via Single Molecule Real-Time (SMRT) sequencing. Mutations introduced to the M150B genome were identified by sequence comparison to the wild-type and published closed sequences. A major mutation (a deletion) in the gene of the master transcriptional regulator of sporulation, Spo0A, was identified. A *spo0A* single gene knockout strain was constructed using a double--crossover genome-editing method. The Spo0A-deficient strain showed similar tolerance to crude glycerol as the evolved mutant strain M150B. Methylation patterns on genomic DNA identified by SMRT sequencing were used to transform plasmid DNA to overcome the native *C. pasteurianum* restriction endonuclease.

**Conclusions:**

Solvent production in the absence of Spo0A shows *C. pasteurianum* differs in solvent-production regulation compared to other solventogenic *Clostridium*. Growth-associated butanol production shows *C. pasteurianum* to be an attractive option for further engineering as it may prove a better candidate for butanol production through continuous fermentation.

**Electronic supplementary material:**

The online version of this article (doi:10.1186/s13068-015-0408-7) contains supplementary material, which is available to authorized users.

## Background

The production of biodiesel has led to production of large quantities of waste glycerol [1]. The transesterification of plant- and animal-derived triglycerides with methanol yields 10 wt% crude glycerol. Crude glycerol can be refined, but increased production of biodiesel has flooded the market, making such processes economically unviable [2]; this economic shift has moved glycerol from coproduct to waste [3]. Conversely, the excess crude glycerol makes it an ideal feedstock for producing chemicals and biofuels. Crude glycerol has been utilized in various ways including as a supplement of livestock diets, as a reactant for chemical catalytic processes, and as a feedstock for chemicals produced by bioconversion [4].

*Clostridium pasteurianum* (*Cpa*), a Gram^+^ Firmicute obligate anaerobe, is capable of making *n*-butanol and 1,3-propanediol (PDO) directly from glycerol as sole carbon source, a capability not shared by other well-studied *Clostridium* organisms [5–7]. Butanol is of particular interest as it is fungible with gasoline due to its high energy density, low vapor pressure, and low water solubility (77 g/L). As glycerol is a more reduced carbon source than glucose, the theoretical yield for butanol production from glycerol is 17 % higher than from glucose on a carbon-mole basis. This reducing power is released in the initial step of glycerol degradation in the form of NADH. *Cpa* has been shown to produce up to 17 g/L of butanol in optimized batch culture [8]. In a more recent study, a mutant *Cpa* strain was shown to produce up to 17.8 g/L butanol from pure glycerol at very high rates from high-density cultures, with minimal byproduct formation [9].

The impurities of crude glycerol are inhibitory to microbial growth [1, 2, 10–12], and are thus a large obstacle to using crude glycerol for producing fuels and chemicals. Fatty acids, salts, and alcohol from biodiesel production persist in significant amounts in crude glycerol. Growth on 25 g/L crude glycerol has been shown to lead to a 40 % decrease in solvent production [2]. In addition, increased lag times and decreased substrate uptake have been reported for several *Clostridium* organisms when grown on crude glycerol [2, 13]. The most toxic of these impurities is linoleic acid, a polyunsaturated omega-6 fatty acid [12]. The nonlinear structure caused by the two double bonds likely causes membrane depolarization [14]. *Cpa* solvent production has been shown to be completely abolished at 1.25 g/L linoleic acid (along with severely inhibited growth), whereas production of solvents was not greatly affected by fatty acids with lower levels of saturation [12]. In order for crude glycerol to be effectively converted to butanol, a tolerant strain of *Cpa* must be developed.

The *Cpa* genome has recently been sequenced and shown to be amenable to genome editing [11, 15], but low transformation efficiencies prohibit the use of high throughput library-based genome engineering tools. Here, we employed directed evolution of mutagen-treated *Cpa* aiming to select mutants tolerant to and using increasing concentrations of crude glycerol as the primary carbon and energy source. A tolerant mutant was isolated which exhibited increased tolerance to crude glycerol and improved butanol productivity. Single Molecule Real-Time (SMRT) sequencing of the mutant and WT strains identified all mutations in the tolerant mutant. Among other variants, a 24-bp deletion was identified in the key sporulation transcriptional regulator Spo0A. We confirm the that Spo0A inactivation is responsible for the tolerant and improved solvent-producing phenotype; we knocked out the *spo0A* gene in the WT strain and show that the engineered strain displays similar tolerance to crude glycerol as the evolved strain. In order to interpret the impact of Spo0A knockout on product formation, we used the *Cpa* genome sequencing to identify and map key product-formation genes, and analyzed them in a comparative analysis against other solventogenic *Clostridium* organisms. SMRT analysis enabled the identification of multiple DNA methylation motifs, some novel, including the confirmation of a cytosine methylation motif which enables the evasion of the endogenous type II endonuclease. We used a codon-optimized version of the native DNA methylase to enable efficient plasmid DNA transformation.

## Results

### Mutation, selection, and isolation of a mutant strain tolerant to crude glycerol

**Fig. 1:**
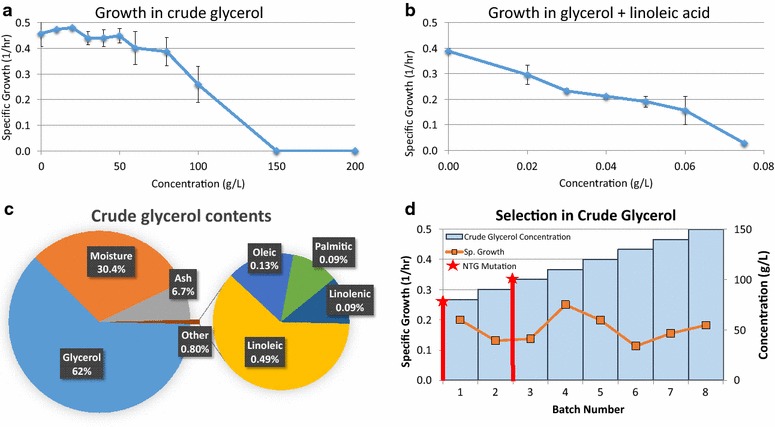
Growth characteristics of WT *C. pasteurianum* in CGM with crude glycerol and linoleic acid and directed evolution selection. **a** WT *Cpa*-specific growth rate as a function of crude glycerol concentration. *Error bars* represent standard deviation, four biological replicates. **b** WT *Cpa*-specific growth rate in 30 g/L pure glycerol as a function of sodium linoleate concentration. *Error bars* represent standard deviation, two biological replicates. **c** Crude glycerol contents by wt%. **d** Selection of mutagenized *Cpa* on crude glycerol. Serial transfers in CGM with the increasing levels of crude glycerol (*blue bars*). The population was mutagenized twice (*red bar* and *star*) and allowed to grow over 8 batches where the population-specific growth rate (*orange squares*) increased in the highest concentration of crude glycerol

Industrial butanol production from crude glycerol would require a strain that can readily grow on crude glycerol with a high growth rate and short lag phase and that can produce high butanol titers with good selectivity. The negative effect of crude glycerol on the growth of the WT strain is apparent at 80 g/L (Fig. 1a), while no growth was observed at 150 g/L or higher. It has been shown that the most toxic components of crude glycerol are the residual fatty acids that persist in the glycerol layer in biodiesel production [12]. The most toxic of these was found to be those with higher degrees of unsaturation, and notably, linoleic acid, which completely abolishes growth of *Cpa* at 1.25 g/L [12] and has been found to negatively affect growth rates of some Gram^+^ bacteria at concentrations as low as 3 mg/L [16]. Here, we found growth of *Cpa* was almost completely abolished at concentrations of 0.075 g/L when grown on 30 g/L glycerol and significantly affected at just 0.02 g/L 9-cis,12-cis-linoleic acid (Fig. 1b).

The crude glycerol (Feed Energy, Des Moines, Iowa) used in this study was analyzed for its respective components (by New Jersey Feed Lab). 0.8–2 % of the crude glycerol is fatty acids. The variation in concentration of fatty acids in the crude glycerol depends on the depth of the sample (low at the bottom, high at the top). As we attempted to uniformly sample from the top of the glycerol for each study, variations are to be expected from batch to batch and especially over time as the crude glycerol stock was depleted. The fatty acid profile of the crude glycerol was over half attributed to linoleic acid (Fig. 1c).

In order to obtain a strain of *Cpa* that is tolerant to crude glycerol, a mutagenesis and directed evolution strategy was employed. *Cpa* cultures were mutated twice by exposure to 50 µg/mL *N*-methyl-*N*′-nitro-*N*-nitrosoguanidine (NTG) for 15 min, washed thrice with PT buffer, and recovered in 2xYTG media as described [17]. The mutated *Cpa* population was grown on CGM-crude glycerol medium and serially transferred eight times in increasing concentrations of crude glycerol (Fig. 1d). Each batch increased in crude glycerol by 10 g/L starting with 80 g/L in batch 1. After 10 days and 28 generations of growth, the final population sustained strong growth with little lag time in 150 g/L crude glycerol, a level that completely inhibited growth of the WT strain. Single colonies from the final population were isolated from solid media and assayed for growth in 100 g/L crude glycerol. A single mutant (M150B) was identified with consistently short lag-phase times.

**Fig. 2:**
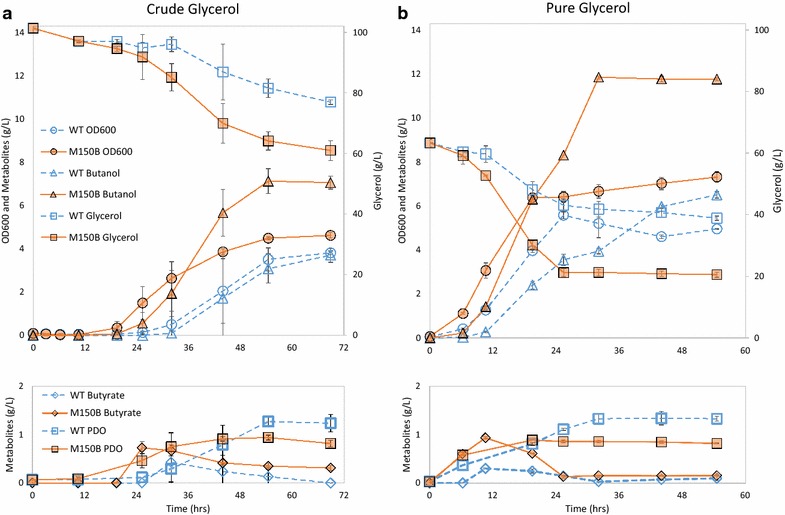
Growth characteristics and metabolite profiles of WT and M150B. Batch cultures of WT (*blue hollow shapes*) and M150B (*orange filled shapes*) were grown in CGM with either 100 g/L crude glycerol (**a**) or 60 g/L pure glycerol (**b**). Turbidity was monitored (OD_600_, *circles*) as well as glycerol (*squares*, *secondary axis*), butanol (*triangles*), butyrate (*lower plot*, *diamonds*), and 1,3 propanediol (*lower plot*, *squares*). *Error bars* represent standard deviation, two biological replicates

In simple batch cultures without pH control and feeding or medium optimization, *Cpa* strain M150B showed a decreased lag phase, 65 % higher glycerol consumption (40.4 g/L), and 91 % higher butanol production (final titer 7.1 g/L) compared to the WT in 100 g/L crude glycerol (Fig. 2a). The WT strain produced more PDO than the mutant (2.6 vs. 1.5 g/L). Interestingly, M150B also outperformed the WT strain when grown on pure glycerol (Fig. 2). While there was no lag phase for either the mutant or WT when grown on 60 g/L pure glycerol, M150B consumed 75 % more glycerol (43 g/L) and produced 80 % more butanol (11.7 g/L). As under the crude glycerol condition, the WT produced more PDO than the mutant from pure glycerol, but for both strains, less was produced overall (1.5 and 0.9 g/L). Acetate, a component of CGM media (~1.8 g/L), was partially consumed by both strains under both conditions. Ethanol and lactate were produced at low levels (<0.5 g/L) in both strains under both conditions.

In addition to the improved fermentation properties, we also observed that this mutant was asporogenous. We confirmed that the M150B strain did not form spores by phase-contrast microscopy after 6 days of growth in CGM media (Additional file 1: Figure S1). We observe plentiful phase-bright sporulation in the WT cells, while the M150B cells show no such spore formation. We also performed a chloroform-based sporulation assay [18] where the WT strain survived 10 min of 50 % v/v chloroform treatment, while M150B did not.

### SMRT sequencing of *Cpa* ATCC 6013 and the M150B mutant strains identifies a large deletion on the *spo0A* gene as likely responsible for the tolerant and butanol-production phenotype

We desired to determine the genetic source of the improved characteristics of the evolved strain M150B. Three *Cpa* ATCC 6013 sequences have been published recently. Each successive genomic sequencing project has resolved the size of the genome and the number of contigs (from 4,285,687 bases assembled into 37 contigs [19], to 4,420,124 bp in 12 contigs [15], to finally 4,351,223 bp in a single closed circular chromosome [20]). All sequence comparisons, gene names, and genomic loci here are based on the closed circular chromosome (Genbank CP009267.1).

We sequenced both the WT and mutated strains using the Pacific Biosciences RSII. The WT genomic DNA was isolated during the transition phase, while the mutant M150B’s gDNA was isolated during mid-exponential phase. The WT sequencing resulted in 46,455 mapped reads with an average length of 8271 bp and an average 78.4× coverage. The M150B sequencing resulted in 107,963 mapped reads with average read length of 6471 and average 121.1× coverage. The coverage of the M150B genome depended on genome locus, while that of the WT did not (Additional file 1: Supplemental Results, Figure S2); the coverage for M150B was the greatest near the origin of replication. We suspect this is due to the state of growth of the culture at the time of gDNA harvesting.

**Fig. 3:**
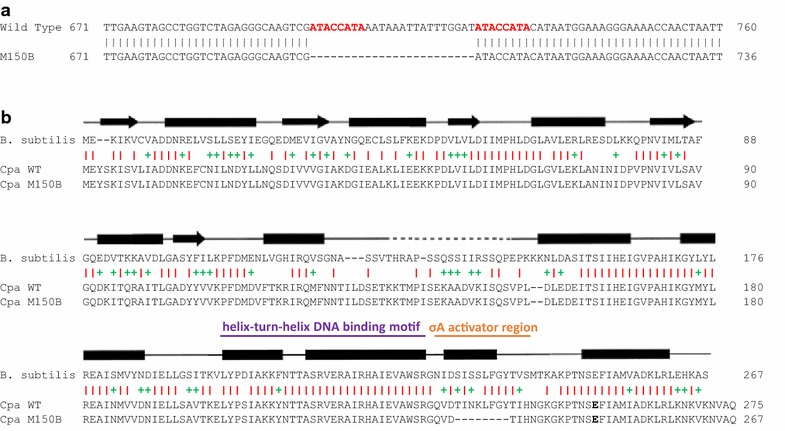
Sequence alignments of Spo0A. **a** Nucleotide sequence alignment of WT and M150B Spo0A region of interest. Nucleotides are numbered from the beginning of the gene. The 8 bp repeat surrounding the deleted region is *bold* and in *red*. **b** Amino acid residue alignment of the well-studied *Bacillus subtilis* (*Bsu*) against *Cpa* WT and M150B. Secondary structures based on *Bsu* from Lewis et al. [25] show conserved beta sheets (*arrows*) and alpha helices (*bars*) as well as the linker region (*dashed line*). Similarity is shown by *red bars* (identity) or green plus symbols (conservative). Helix-turn-helix DNA-binding motif is indicated by a purple bar, and the σ^A^-activating region is shown with an *orange bar*

We compared our sequence of the WT ATCC 6013 strain with the most recently published sequence [20] and observed 17 differences (Additional file 1: Table S1). We did not observe the mutation noted in the Spo0A gene by Rotta et al. [20]. In contrast to the reported sequence, position 255 of Spo0A is glutamic acid, not the lysine (or glutamine) reported for ATCC 6013, making it similar to the *Cpa* DSM 525 [21] sequence and other *Clostridium* strains. When comparing the sequence of M150B with the same published sequence, we found the same 17 differences contained in our WT strain along with 67 other variants (Table 1). Of these 67 variants, 66 were substitutions. Ten were in regions that were not annotated to contain genes. Of the 56 remaining, 14 were silent mutations, 14 were conservative amino acid substitutions, one generated a premature stop codon in a putative glycosyltransferase, and 27 others were nonconservative amino acid substitutions. In addition, we found a single deletion of 24 bp in the gene encoding the master sporulation factor Spo0A [22] (Fig. 3).

**Table 1:** Sequence variants from sequenced wild-type *Cpa* 6013 vs. M150B

Mut. locus [20]	Mut. type	CDS	Mut.	Ref.	Var.
135360	Sub.	No CDS		A	G
177873	Sub.	Hypothetical cell wall-binding protein, *c01710*	A267V	C	T
256066	Sub.	Glycosyltransferase, *c02430*	I257T	A	G
328268	Sub.	Xanthine dehydrogenase, molybdenum binding; *pudD1*	P483L	C	T
511135	Sub.	Pyridine nucleotide-disulfide oxidoreductase; NADH dehydrogenase; coenzyme A disulfide reductase; *c04890*	P187S	T	C
547010	Sub.	Pyruvate-flavodoxin oxidoreductase; *nifJ1*	S579N+	G	A
609918	Sub.	Lactose permease; *lacS*	P429S	G	A
623217	Sub.	lysin; *c05770*	A129T	G	A
633860	Sub.	Hypothetical protein; *c05850*	Silent	C	T
662676	Sub.	High-affinity nickel-transport protein; *nixA1*	Silent	C	T
664502	Sub.	Methyl-accepting chemotaxis protein; *c06130*	T311A	A	G
774540	Sub.	Metallophosphoesterase; *c07050*	G294E	G	A
805746	Sub.	Putative amidase domain-containing protein; *c07320*	G378E	G	A
822080	Sub.	tRN Glu)-specific nuclease WapA; *wapA1*	S1026F	C	T
849186	Sub.	Putative glycosyltransferase; *c07760*	Q641*tag*	G	A
888969	Sub.	Phage infection protein; *c08050*	E95K+	C	T
905016	Sub.	putative L-ascorbate-6-phosphate lactonase UlaG; *ulaG*	A128V	C	T
959280	Sub.	No CDS		C	T
1010600	Sub.	Radical SAM domain protein; *c09370*	G32D	C	T
1018923	Sub.	No CDS		G	A
1034853	Sub.	Putative AAA-ATPase; *c09620*	E311K+	G	A
1047038	Sub.	DNA-binding response regulator; *c09780*	Silent	G	A
1052341	Sub.	Hypothetical protein; *c09830*	E98K+	G	A
1058795	Sub.	Nicotinamidase; *c09830*	V31I+	G	A
1263298	Sub.	No CDS		G	A
1384612	Sub.	Spore germination protein KA; *gerKA2*	G15D	G	A
1408727	Sub.	hypothetical protein; *c13080*	L71N	G	A
1595471	Sub.	Lactate-responsive regulator LldR; *c14940*	A154T	G	A
1597713	Sub.	Electron transfer flavoprotein, alpha subunit; *etfA1*	A300T	G	A
1608597	Sub.	CAAX amino terminal protease family protein; *c15040*	Silent	T	C
1729728	Sub.	Integral membrane protein; *c16180*	V8I+	G	A
1796914	Sub.	Phenylalanyl-tRNA synthetase beta chain; *pheT*	A674V	C	T
1957368	Sub.	Flagellum-specific ATP synthase FliI; *fliI*	E44K+	G	A
1973896	Sub.	RNA polymerase sigma factor for flagellar operon; *whiG*	A75T	G	A
1976216	Sub.	Flagellar basal-body rod protein; *flgG3*	Silent	T	C
2055679	Del.	Stage 0 sporulation protein A; *spo0A*	Del. 235–242	ATACCATAAATAAATTATTTGGAT	
2822361	Sub.	CRISPR-associated helicase/endonuclease Cas3; *cas3*	E316F	C	T
3198907	Sub.	No CDS		C	T
3208358	Sub.	Exonuclease SbcC; *sbcC*	A558T	C	T
3222314	Sub.	Hypothetical protein, CF-17 family; *c29890*	E213K+	C	T
3270753	Sub.	No CDS		C	T
3272757	Sub.	tRNA-dihydrouridine synthase; *c30410*	D126K+	C	T
3273913	Sub.	Fe-S oxidoreductase; *c30430*	T87A	A	G
3297381	Sub.	No CDS		G	A
3348577	Sub.	Transcriptional regulator, TetR family; *c31160*	S20N+	C	T
3399757	Sub.	Hydrolase (HAD superfamily); *c31590*	P219S	G	A
3439637	Sub.	Multiple sugar transporter, membrane-spanning permease protein MsmG; *msmG2*	A236V	G	A
3488562	Sub.	Potassium-transporting ATPase C chain; *kdpC*	T283I	G	A
3497476	Sub.	Fatty-acid peroxygenase; *cypC*	P255S	G	A
3521979	Sub.	ATP phosphoribosyltransferase regulatory subunit; *hisZ*	P96L	G	A
3531067	Sub.	Cysteine desulfurase; *c32790*	Silent	G	A
3535341	Sub.	Chaperone protein HtpG; *htpG*	Silent	G	A
3593919	Sub.	No CDS		G	A
3595710	Sub.	2-iminoacetate synthase; *thiH2*	P77S	G	A
3641811	Sub.	Possible surface protein; *c33730*	T742I	G	A
3666696	Sub.	No CDS		G	A
3698846	Sub.	L-arabinose transport system permease; *araQ2*	T235I	G	A
3759007	Sub.	hypothetical protein; *c34910*	E729K+	C	T
3776313	Sub.	RNA-binding protein; *c35120*	S523N+	C	T
3863797	Sub.	DNA-3-methyladenine glycosylase II; *c35950*	E119K+	C	T
3872706	Sub.	Autoinducer 2 sensor kinase/phosphatase LuxQ; *luxQ3*	E242F	C	T
3909641	Sub.	Signal-transduction and transcriptional-control protein; *stc3*	A657V+	C	T
3935380	Sub.	50S ribosomal protein L2; *rplB*	Silent	C	T
3936650	Sub.	50S ribosomal protein L3; *rplC*	G191D	C	T
4163498	Sub.	Signal transduction histidine kinase; *c39070*	S439N	C	T
4231311	Sub.	Putative competence-damage inducible protein; *cinA*	Silent	C	T

Positive substitutions are indicated by a plus symbol

We also observed three point mutations in flagella-related genes. A silent mutation was found in *flgG3*, coding for a flagellar protein and a conservative mutation was found in the flagellum-specific ATP synthase, *fliI* (E44K). A nonconservative mutation was found in *whiG*, coding for a sigma factor specific to a flagellar operon (Table 1). The A75T mutation in the WhiG gene is in a highly conserved region in WhiG and σ^D^ proteins supposed to be the promoter recognition element [23]. The combined effects of these mutations may be responsible for M150B not staying in suspension as long as its parent strain. M150B cultures consistently settle quickly and more compactly compared to the WT when not actively agitated (Additional file 1: Figure S3).

**Fig. 4:**
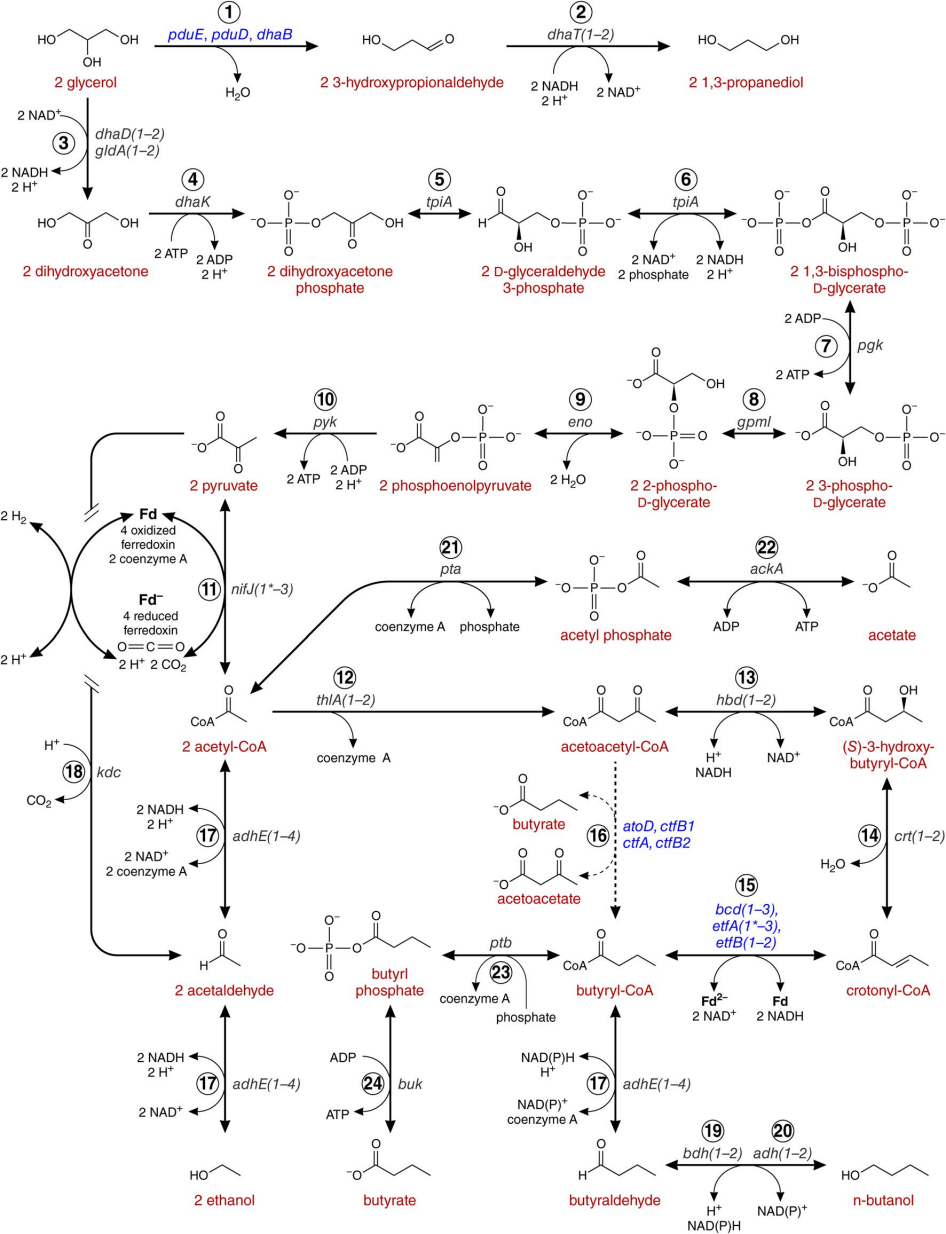
Genetic basis of solventogenesis from glycerol in *C. pasteurianum*. Possible metabolic pathways for glycerol to 1,3 propanediol, ethanol and butanol. Gene names coding for the enzymes catalyzing the corresponding reactions are listed in italics. Multiple genes coding for a complexed functional protein are *colored blue*. Paralogs annotated with the same function and name are noted in *parentheses*. *Asterisks* indicate genes found mutated in M150B. Enzyme names are as follows:* 1* glycerol dehydratase, *2* 1,3-propanediol dehydrogenase, *3* glycerol dehydrogenase, *4* dihydroxyacetone kinase, *5* triosephosphate isomerase, *6* glyceraldehyde-3-phosphate dehydrogenase, *7* phosphoglycerate kinase, *8* phosphoglycerate mutase, *9* enolase, *10* pyruvate kinase, *11* pyruvate-flavodoxin oxidoreductase, *12* acetyl-CoA acetyltransferase, *13* 3-hydroxybutyryl-CoA dehydrogenase, *14* 3-hydroxybutyryl-CoA dehydratase, *15* butyryl-CoA dehydrogenase/Etf complex, *16* butyrate-acetoacetate CoA-transferase, *17* aldehyde-alcohol dehydrogenase, *18* possible pyruvate decarboxylase, *19* NADH-dependent butanol dehydrogenase, *20* NADPH-dependent butanol dehydrogenase, *21* phosphate acetyltransferase, *22* acetate kinase, *23* phosphate butyryltransferase, *24* butyrate kinase

**Fig. 5:**
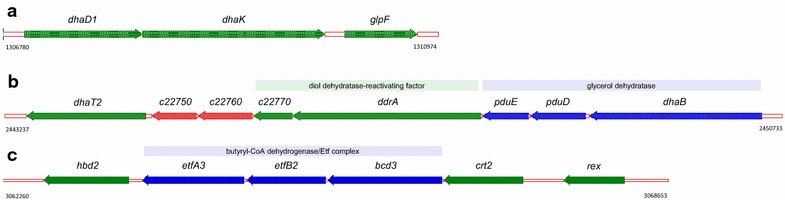
**a**–**c** Cartoons of parts of the glycerol to butanol genomic loci: **a** glycerol to central metabolism, **b** reductive pathway of glycerol to 1,3-propanediol (*c22750* codes a hypothetical protein and *c22760* codes for cobyrinic acid a,c-diamide adenosyltransferase), **c** acetoacetyl-CoA to butanoyl-CoA locus including the Etf/Bcd complex (*rex* encodes a redox-sensing transcriptional repressor)

We also observed point mutations in genes related to central metabolism and solvent production. *nifJ1* encoding pyruvate–flavodoxin oxidoreductase, which catalyzes pyruvate and coenzyme A to acetyl-CoA and carbon dioxide (Fig. 4) has a S579 N mutation. However, *Cpa* contains two paralogs, *nifJ2* and *nifJ3*, which may compensate for the mutated *nifJ1*. The *etfA1* gene, encoding the electron-transfer flavoprotein alpha subunit, which contains an FAD-binding domain, has an A300T mutation. The *Cpa* genome encodes the paralog *etfA3*, which contains the major domains of the EtfA (Fig. 5c). EtfA together with EtfB and butyryl-CoA dehydrogenase likely form the Etf/Bcd complex, which couples the NADH-dependent reduction of crotonyl-CoA to butanoyl-CoA with the reduction of ferredoxin [24] (Fig. 4).

The deletion of 24 bp in M150B’s single copy of the *spo0A* gene led to an in frame deletion of eight amino acid residues in the σ^A^ activator region (Fig. 3) [25]. The σ^A^ activator region is essential for binding to σ^A^-dependent promoters [25]. This mutation is unique in that it is a deletion and not an A-G or T-C single nucleotide variant, commonly observed in NTG-mutated strains. We observed that the deletion of the 24 bp corresponded exactly with two identical 8 bp sequences (5′-ATACCATA-3′) surrounding the deletion (Fig. 3). This is likely due to homologous recombination of these overlapping regions, which removed the section between these sequences and one of the copies of the sequence. It is noteworthy that while sporulation was disrupted as could be predicted by such a deletion, solvent production remained. This is unexpected as it stands in contrast to what was observed in Spo0A mutants in *C. beijerinckii* (*Cbe*) [26] and *C. acetobutylicum* (*Cac*) [22]. In those strains, Spo0A regulates the expression of key solvent genes and notably of the genes in the *sol* locus [22], and as a result, in their Spo0A mutants, solvent production is nearly abolished.

### Mapping the solvent-formation capabilities of *C. pasteurianum* on its sequenced genome illuminates the role of multiple gene orthologs/paralogs on its unique metabolic traits

In order to gain a better understanding of the aforementioned differences in the regulation of solvent formation between *Cpa* and the two well-established model solventogenic *Clostridium* organisms, we mapped the core primary metabolic reactions of *Cpa* to the sequenced genome and compared this mapping to that in *Cac*. This has only recently been enabled through a closed and annotated genome sequence.

Glycerol utilization by the cells leads to either entry to glycolysis at dihydroxyacetone phosphate or reduction to form 1,3-propanediol (PDO) (Fig. 4). The genes responsible for glycerol oxidation pathway into glycolysis, glycerol dehydrogenase and dihydroxyacetone kinase (*dhaD1*, c12150 and *dhaK*, c12160), are sequentially located in a likely operon with the glycerol uptake gene *glpF* (c12170) (Fig. 5a). It should be noted, though, that three additional glycerol dehydrogenase paralogs are located throughout the genome (*gldA1*, c28060, 29 % identical to *dhaD1* by protein; *gldA2*, c33610, 27 %; and *dhaD2*, c38340, 43 %). The genes for the reductive pathway to PDO are also colocated in a likely operon (Fig. 5b). The propanediol dehydratase is encoded by three genes (*pduE*, c22790; *pduD*, c22800; and *dhaB*, c22810), which precede a diol dehydratase-reactivating factor gene (*ddrA*, c22780) and the PDO dehydrogenase (dhaT2, c22740). The PDO dehydrogenase also has an extra copy (*dhaT1*, c11040, 43 % identical by protein). The presence of these multiple gene paralogs suggests their likely importance in achieving the fast rates of glycerol utilization and growth on glycerol.

**Fig. 6:**
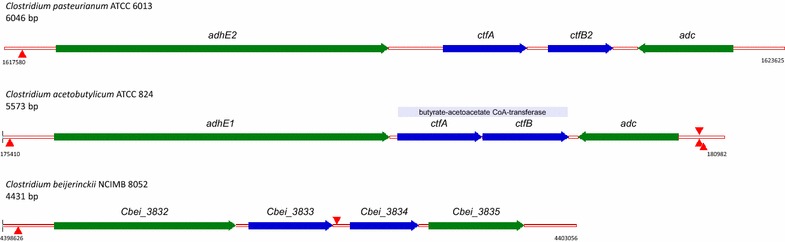
Cartoon of the *sol* loci in *C.pasteurianum*, *C. acetobutylicum*, and *C. beijerinckii*. Genomic positions are given at the beginning and end of the cartoon, and the relative sizes are to scale. Reported and putative 0A boxes are indicated by *red triangles* above the cartoon on the top strand and below if on the bottom strand

**Fig. 7:**
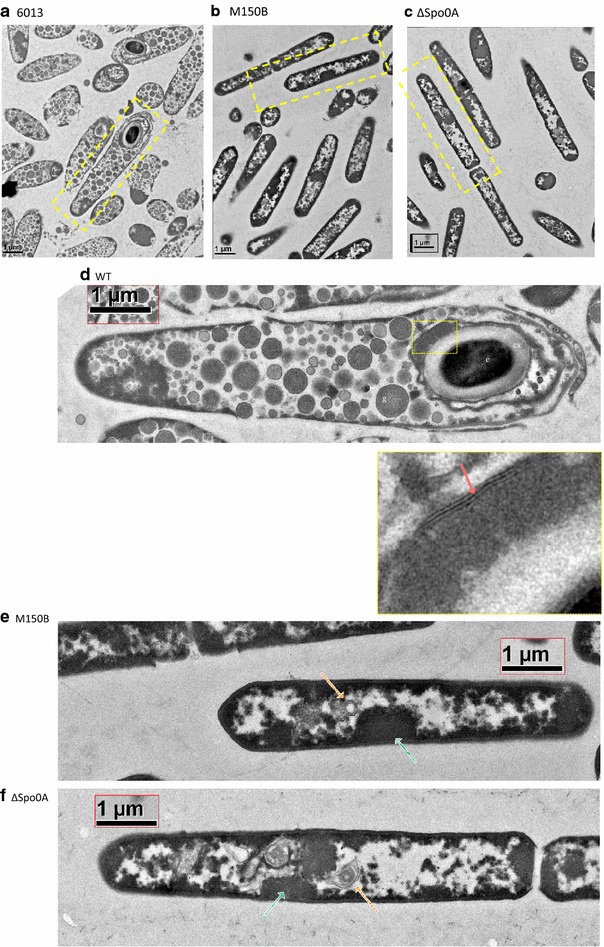
Transmission electron micrographs of *C. pasteurianum* after 5 days in culture. **a** WT cells show characteristic sporulation development including clostridial-form bulging cell shape. **b**–**c** M150B and ΔSpo0A and cells are *rod shaped* with no obvious sporulation development. **d** Isolation and enlargement of individual WT cell image from 7A shows spore core (c), cortex (cx), granulose bodies (g), and the multilayered spore coat structure (*solid red arrow*). **e**, **f** Isolation and enlargement of individual M150B and ΔSpo0A cell images from 7b, c show probable mesosomes (*thick striped arrows*) and globular electron dense regions (*fine striped arrows*)

It has been well established that *Cpa* ferments glycerol to form PDO and butanol (Fig. 2). Unlike the model solventogenic *ClostridiumCac*, it does not produce acetone [19, 27]. It does, however, have a *Cac*-like *sol* locus on its chromosome containing *adhE2* (a fusion protein coding for an aldehyde-alcohol dehydrogenase, c15160), followed by *ctfA* and *ctfB2* (c15170, c15180, encoding the two units of the CoA-transferase, CoAT), and, next, in an opposite orientation a monocistronic putative *adc* (acetoacetate decarboxylase, Adc, c15190) (Figs. 6a, 7). In *Cac*, AdhE1 catalyzes the conversion of butyryl-CoA to butanol [28, 29], CoA-transferase (CoAT) catalyzes the conversion of acetoacetate to acetoacetyl-CoA [30], which is accompanied by acetate and/or butyrate uptake. Acetoacetyl-CoA is converted to acetone by Adc [31]. In *Cac*, the *sol* locus, encoded on the pSOL1 megaplasmid [32], contains the *adhE1*, *ctfA*, *ctfB* genes in an operon organization (although recent work in our lab shows that the *ctfA/B* genes can be also transcribed independently of the *adhE1* gene), and the monocistronic *adc* gene in the opposite orientation. There is exists a very similar paralog of the AdhE1 protein also encoded on the *Cac* pSOL1 megaplasmid, annotated as *adhE* (CA_P0035), but that protein, although it can catalyze butanol formation, is not involved in the in vivo formation of butanol as it is not expressed under normal solventogenic conditions [33]. The *Cpa adhE2* gene is 76 % similar with the *Cac adhE1* on the DNA level and 82 % on the amino acid level. Likewise, the *ctfA*, *ctfB2*, and *adc* genes are similar to their *Cac* orthologs (*Cac ctfA* 74 % by DNA, 73 % by protein; *Cac ctfb* 71 % by DNA, 71 % by protein; *Cac adc* 76 % by DNA, 84 % by protein, respectively). *Cpa* has three more annotated *adhE* genes (*adhE1*, *c05730* [similarities: 73 % DNA, 77 % protein to *CacadhE1*]; *adhE3*, c15660 [70 % DNA, 64 % protein to *Cac adhE*]; and adhE*4*, c28930 [73 % DNA, 76 % protein to *Cac adhE1*]) and two butanol dehydrogenase genes (*bdh1*, c04370 [71 % DNA, 72 % protein to *Cac bdhA*] and *bdh2*, c15980 [46 % DNA, 39 % protein to *CacbdhB*]) located in different parts of the genome. Notably, *Cpa* contains an additional set of genes encoding a CoAT (*atoD*, *c08850* [55 % DNA, 46 % protein to *Cac ctfA*] and *ctfB1*, *c08840* [62 % DNA, 55 % protein to *Cac ctfB*]). One assumes naming c08850 ‘*atoD*’ was inspired by the standard *Escherichiacoli* nomenclature, but it might be more appropriately named *ctfA1* (with the c15170 gene as *ctfA2*). The *kdc* gene (keto-acid decarboxylase) of *Cpa* (c04400) has 53 % similarity to the pyruvate decarboxylase gene of *Cac* (*pdc*, 40 % by protein), which catalyzes the conversion of pyruvate to acetaldehyde.

When comparing the *sol* loci in *Cpa* and *Cac*, we observe relatively large gaps between the *adhE2*, *ctfA*, and *ctfB2* genes in *Cpa* (432 bp and 162 bp, respectively), while the *Cac* orthologs are much closer together (only 63 and 5 bp), indicating a tighter operon structure (Fig. 6). The expression of the *CacadhE1*-*ctfAB* operon has been shown to be controlled by an activated (phosphorylated) Spo0A(34), and a Spo0A-binding site (0A box) has been proposed [26] (Fig. 6). In addition to the Spo0A-binding site, *Cac* has putative repeated regulatory elements, which overlap with the 0A box [34]. While *Cpa* has a similar 0A box in this region, these repeated elements do not seem to be present. In addition, *Cpa* has 3 additional *adhE* genes, two of which do not have any identifiable 0A boxes upstream. This may play a role in butanol formation being unaffected by the loss of Spo0A activity as demonstrated by the inability of the strain to sporulate. *Cac* also has three putative 0A boxes upstream of the *adc* gene. *Cpa* has none that could be identified. This is notable since *Cpa* seems to contain all the genetic elements necessary for acetone production (an intact *sol* locus), it does not produce acetone. This would suggest that the genes for acetone formation may be expressed and used only under specific culture conditions not currently employed in standard laboratory *Cpa* cultures.

The *Cbesol* operon differs considerably from *Cac* and *Cpa* (Fig. 6). Its *sol* operon does not contain a bifunctional *adhE* gene as in *Cac* and *Cpa*, but contains a gene coding for an aldehyde dehydrogenase (*ald*, *Cbei_3832*). A bifunctional *adhE* gene (*Cbei_0305*) is located in another locus. The *adc* gene (*Cbei_3835*) is oriented in the same orientation as *ald*, *ctfA*, and *ctfB* and these are all transcribed in a polycistron [35] with a putative 0A box upstream.

These differences in the regulation of solventigenic genes between *Cpa* and the two classical solventogenic clostridia, *Cac* and *Cbe*, are of fundamental and practical interest. The role of Spo0A in solvent production regulation results in more complex phenotypes, and, notably, strain degeneration and bistability in solvent production [18, 36, 37]. Strain degeneration refers to the simultaneous loss of the ability to sporulate and produce solvents. While bistability in solvent production refers to the different outcomes (high versus low) of solvent production depending on the state of the culture inoculum used. The two phenotypes are linked and are likely related to the complex role of the sporulation-specific sigma factor SigK, which controls both Spo0A expression as well as late sporulation events [36, 38]. *Cpa* appears to be shielded from such phenotypic complexities, which result in performance-limiting practical bioprocessing issues [31].

To sum, mapping of the proteins that are responsible for solvent formation on the *Cpa* genome, and comparative analysis against the corresponding genes in the two well-studied *Clostridium* solventogens, *Cac* and *Cbe*, demonstrated distinct genetic organizations and regulatory elements that would explain the observed phenotypic differences. The latter include the fact that, in contrast to *Cac* and *Cbe*, butanol formation in *Cpa* is not controlled by Spo0A, and is in fact growth associated (Fig. 2) rather than a stationary-phase phenomenon.

### New findings from revisiting the SMRT-sequencing-enabled *Cpa*-DNA methylation analysis

Using SMRT sequencing it is possible to interrogate the native methylome of the genome as no amplification steps occur prior to sequencing. As the DNA is being sequenced, modification to bases result in a delay in the called bases, called the interpulse duration ratio (IPD) [39]. Using the PacBio RS Modification and Motif Analysis (MAMA) protocol, we observe in both the WT and M150B genomes the previously reported *N*^6^-methyladenine modification motifs associated with Type II and Type I restriction modification systems in *Cpa* (m-6A modifications: 5′-GA^m^TC-3′ and 5′- AA^m^GNNNNNCTCC-3′, respectively) [15]. The MAMA protocol also returned two previously unreported *N*^6^-methyladenine modification motifs 5′-GRTAAA^m^G-3′ and 5′-CAAAAA^m^R-3′ in both sequenced genomes (R = G or A; Table 2).

**Table 2:** Methylation data and analysis

Motif	Modified position	Type	% motifs detected	# of motifs detected	# of motifs in genome	Mean modification QV	Mean motif coverage	Partner motif
GRTAAAG	6	m6A	99.65	2579	2588	87.0	55.4	
AAGNNNNNCTCC	2	m6A	99.61	511	513	93.1	59.4	GGAGNNNNNCTT
GGAGNNNNNCTT	3	m6A	99.03	508	513	88.1	58.0	AAGNNNNNCTCC
GATC	2	m6A	99.14	13,348	13,464	85.8	58.4	GATC
CAAAAAR	6	m6A	97.10	3786	3899	74.0	56.3	

Methylcytosine modifications are more difficult to determine compared to m-6A [39]. *Cpa* carries a Type II restriction endonuclease identified as a barrier to plasmid transformation as it recognizes and cuts 5′-CGCG-3′ motifs [11, 40]. The MAMA protocol returned two different motifs containing 5′-CGCG-3′ for the WT and M150B (5′-CGC^m^GNNNANNNTNNANA-3′ and 5′-MNNNC^m^GCGAANANT-3′, respectively), but these seem to be overspecified since there are only 11 and 14 of these sequences in the *Cpa* genome, respectively. Furthermore, the modified base differs between the two. By analyzing the IPD data directly, however, we observed that the average IPD for the first cytosine of 5′-C^m^GCG-3′ (1.25) is significantly higher than the IPD in 5′-C^m^-3′ (0.86) and 5′-C^m^G-3′ (0.89) (two-sided heteroscedastic *t* test *p* values of 9.0E−7 and 4.5E−6, respectively), indicating this motif is likely modified. The IPD for the second cytosine in 5′-CGC^m^G-3′ (1.11) is also significantly higher than in 5′-C^m^-3′ and 5′-C^m^G-3′ (*p* values of 1.2E−3 and 4.1E−3, respectively), but it indicates lesser frequency of methylation on the second cytosine.

We identified *bepIM* (*c05910*) on the *Cpa* genome encoding the modification methylase BepI. We codon optimized the *CpabepIM* for *E. coli* and had it synthesized and cloned into a plasmid (pCpaDcm2.0, Additional file 1: Table S2, DNA 2.0). Prior to transformation, we first methylated all shuttle plasmids by transforming them into an *E. coli* methylation strain carrying pCpaDcm2.0. No successful transformations were performed without this prior in vivo methylation. Plasmid transformation in *Cpa* has been shown previously using the heterologous M.FnuDII methyltransferase (5′-C^m^GCG-3′) to evade the endogenous restriction enzyme [11]. While methylation of plasmids with heterologous genes is effective, methylome analysis and synthetic expression of the native methyltransferase enzymes is a generalizable method to transform bacteria with an unknown endonuclease recognition sequence.

### Construction of *Cpa* Spo0A knockout confirms that Spo0A inactivation is responsible for conferring tolerance to and better growth on crude glycerol, as well as enhanced growth-associated butanol production

The mutation in *spo0A* was the most conspicuous of all identified, so we decided to test the hypothesis that this deletion was the primary cause of the changes in phenotype we observed. We used the MazF knockout system [41] to design a 200 bp deletion at the start of the *spo0A* gene, including the ribosomal-binding site, start codon, and 60 codons. The design replaces this 200 bp with a chloramphenicol acetyltransferase gene (*cat*) with FRT sites on either side. The recombination plasmid (pKOmazF-Spo0A) contains regions of homology (sized 536 and 506 bp) flanking either side of the *cat* gene and FRT sites. The plasmid was constructed in the manner as previously described [41] and transformed via the previously reported protocol [11] with the exception of the methylation method (see above). Successful recombination was confirmed via size verification of the insert via PCR and agarose gel imaging from three isolated colonies that grew on solid 2xYTG supplemented with thiamphenicol and lactose. Recombination plasmid removal was performed with serial transfers in unselective media and confirmed by replica plating. The integrated *cat* gene was removed with the transformed p94-FLP containing the flippase gene as described [41] and confirmed by PCR and Sanger sequencing. Briefly, *CpaΔspo0A::ThR* + p94-Flp was grown in liquid media supplemented with erythromycin at 30 °C and subsequently plated on solid 2xYTG + Em media. Replica plating confirmed thiamphenicol sensitivity. Loss of p94-Flp was confirmed by replica plating after two serial transfers in unselective media.

To confirm the sporulation deficient phenotype was due to the deletion in the *spo0A* gene, the WT, M150B, and ΔSpo0A strains were imaged by transmission electron microscopy (TEM). The three strains were cultured for 5 days before cells were prepared for TEM analysis. As expected, the WT strain (Fig. 7a, d) displayed the characteristic features of sporulating solventogenic *Clostridium* cells (and notably of *Cac* cells [18, 42]), showing spore development at different maturation stages, and an unusually large number of the characteristic granulose bodies for all cells compared to other *Clostridium* organisms. The granulose bodies appear as lightly electron dense in an electron translucent background, which is also somewhat unusual compared to other solventogenic *Clostridium* cells.

The M150B and ΔSpo0A strains display no granulose bodies, septum, or forespore structures, showing only vegetative cells (Fig. 7b, c) with no bulging structures characteristic of sporulating clostridial-form cells [18, 42]. All cells displayed in their core irregular electron translucent regions, many cells contain one or two globular lightly electron dense features, all surrounded by an electron dense region that defines the cell shape (Fig. 7e, f). We also observed identifiable membranous structures (Fig. 7e, f), which may be mesosomes, an artifact of the chemical fixation process [43]. The M150B and ΔSpo0A cells look similar to the ΔsigK *Cac* cells [38], where sporulation has been blocked just prior to Spo0A expression and activation, and also similar to the *Cac* cells with an inactivated *sigF* gene [37]. In *Clostridium* organisms [36], SigK acts both very early (prior to Spo0A expression) and very late in sporulation, while SigF acts just after Spo0A activation.

**Fig. 8:**
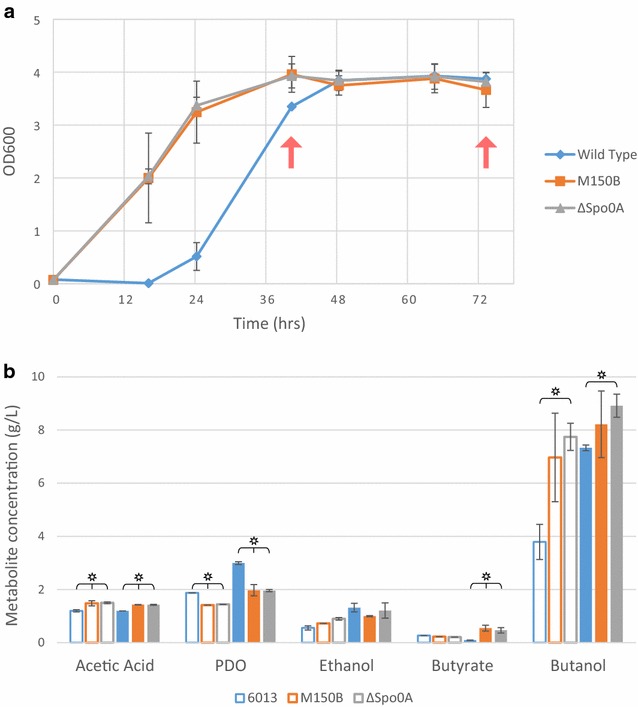
Growth and metabolite analysis of *C. pasteurianum* WT, M150B, and ΔSpo0A strains in CGM with 100 g/L crude glycerol. **a** OD_600_ shows extended lag phase of WT (*blue diamonds*) compared to M150B (*orange squares*) and ΔSpo0A (*gray triangles*). *Red arrows* indicate samples taken for analysis. **b** Metabolite profiles at 40 h (*hollow*) and 73 h (*solid*) of WT (*blue*), M150B (*orange*), and ΔSpo0A (*gray*). *Error bars* represent standard deviation, two biological replicates. Stars indicate *t* test *p* value <0.05 between WT and either or both M150B and ΔSpo0A. Note that the initial CGM media contains 1.8 g/L acetic acid

We tested the growth of the knockout strain in 100 g/L crude glycerol along with the wild-type and the M150B mutant. The M150B and ΔSpo0A strains show a markedly reduced lag time compared to the WT strain (Fig. 8a). Unlike previous results (Fig. 2), the WT strain achieves similar OD to the evolved strain after 48 h. Metabolite analysis by HPLC show improved butanol formation for the Spo0A knockout strain (Fig. 8b). The WT strain produced 50 % more PDO than the M150B and ΔSpo0A strain, which is consistent with what was seen previously. These data show that the observed phenotype of the M150B strain in tolerating higher levels of crude glycerol, faster growth and reduced lag time on glycerol and improved levels of butanol formation can be recapitulated by Spo0A inactivation.

## Discussion

It has been suggested that *Cpa*’s ability to consume glycerol as the sole carbon source is likely due to its reductive PDO pathway. As glycerol is more reduced than glucose, more reducing equivalents are generated to get to glyceraldehyde-3-phosphate, necessitating some glycerol reduction to PDO [8]. The *Cac* genome does not contain the PDO producing pathway, which is likely the reason it cannot use glycerol as a sole substrate [7].

*Cpa* contains multiple paralogs of genes in the glycerol to butanol pathway: glycerol dehydrogenase (4 annotated paralogs), aldehyde-alcohol dehydrogenase (*adhE*) [4], pyruvate-flavodoxin oxidoreductase [3], acetoaceate CoA-transferase [2], 1,3-propanediol dehydrogenase [2], NADH-dependent butanol dehydrogenase [2], and NADPH-dependent butanol dehydrogenase [2]. The large number of paralogs of these core genes may account for the observed high rates of growth and product formation. It has been noted that *Cpa* fermentation products vary under similar conditions, and was thus theorized that the regulation of fermentation related genes is not very strict [8].

Using whole-genome sequencing we are able to fully assess the differences between the parent and evolved daughter strain, M150B. We showed that inactivation of the master regulator of sporulation in *C. pasteurianum*, and all *Clostridium* organisms [36], Spo0A, confers an increased tolerance to crude glycerol stress. As M150B was superior to WT in pure glycerol as well, another selection mechanism seems to have been fast growth on glycerol. Spo0A is a highly conserved protein, which is the master regulator of sporulation in *Clostridium* and *Bacillus* [36, 44]. Spo0A comprises a phosphoacceptor domain and a transcription activation, or ‘effector’, domain. Phosphorylation of the phosphoacceptor domain affects transcription, both positively and negatively, for a large number of genes by binding to Spo0A-binding motif (the ‘0A box’, 5′-TGNCGAA-3′) [25, 26]. The 0A box can be found in the promoter region of a variety of stationary-phase-related genes controlled by the activated (phosphorylated) Spo0A, including, in *Clostridium* organisms, the gene for the first sporulation-specific sigma factor SigF [36]. Blocking the expression of SigF blocks the expression of the all downstream sporulation-specific sigma factors (SigG, E and K), and this is the mechanism by which Spo0A inactivation abolishes sporulation [36].

The effector domain contains a DNA-binding helix-turn-helix region along with a σ^A^ activator region (Fig. 3) [25]. Mutations of *B. subtilis*’ *spo0A* in this region have been shown to have lower or failed activation from σ^A^ promoters and these mutants are asporogenous [25]. The activation of Spo0A in *Cac* has been shown to be dependent on one of two histidine kinase mediated phosphorylation pathways: one involving a single gene *Cac_0323* (having 32 % identical ortholog *Cpa_c33080*) and the other necessarily involving both the genes *Cac_0903* and *Cac_3319* (with 38 and 27 % identical orthologs *Cpa_c33080* and *Cpa_c19640*, respectively) [45]. A *Cac_3319* mutant showed significantly reduced sporulation activity as well as increased butanol production [45, 46], showing solvent production regulation to be a highly complex and relatively poorly understood process.

The presence of Spo0A has been shown to be essential for solventogenesis in *Cac* and *C. beijerinckii* [26]. Overexpression in *Cac* leads to early onset of sporulation and solventogenesis [22], as well as to increased butanol tolerance and prolonged metabolism as a result of large and complex changes in the transcriptional program of the cells [47]. Strong butanol production in the *Cpa* ΔSpo0A shows that although *Cpa* is closely related to *Cac*, solvent production is not regulated in the same manner. Beyond the present findings that demonstrate that Spo0A is not necessary for butanol formation, there are also several other distinct differences worth noting. First, butanol formation in *Cpa* is growth associated in contrast to *Cac* and *Cbe,* where butanol production occurs as active growth ceases and continues into the stationary phase of the culture [42]. Since little butyrate and no acetate is formed in *Cpa* fermentations (Fig. 2), acid and notably butyrate [48] (or butyryl-P [49]) is not obviously a trigger for solventogenesis as in *Cac* and other solventogenic *Clostridium* organisms. Related to the latter is the fact that, as extensively discussed [9], in *Cpa*, there is no acetone formation, which characterizes solvent formation in *Cac* and *Cbe,* where it accompanies acid re-uptake. It is still a mystery why *Cpa* produces no acetone despite the fact that, as discussed above, all the genes for its formation are present on the *Cpa* genome, some with several paralogs. In this context, it is noteworthy that *Cac,* when grown on glucose in the presence of glycerol in continuous culture, it forms no acetone [7]. The authors of Ref. [7] noted that this is mechanistically similar to the impact of gassing continuous culture of *Cac* with carbon monoxide [50], which inhibits hydrogen production. They concluded [7] that: “Acetone formation requires no reduction energy, and it appears that the cellular control mechanisms avoid its production in order to maximize the regeneration of NAD(P) in the reduced nucleotide-consuming pathway”.

## Conclusion

In conclusion, this present study provides a genomic understanding of the unique phenotypic *Cpa* characteristics, and strengthens the case for using *Cpa* as a butanol producer. It has the advantage of growth-associated butanol production with little concomitant acid and no acetone production. Growth-associated butanol production would in principle enable the development of continuous or continuous-like processes, which are difficult to develop with the well-known solventogens like *Cac* and *Cbe* due to the well-known degeneration phenotype [31, 36]. As was shown recently [9], *Cpa* strains and culture media can be readily developed to enable *Cpa* cultures of high cell densities producing high titers of butanol with very good productivities. The present finding would further increase the appeal of such *Cpa* strains, which can become even more productive with further strain engineering, such as for minimizing or eliminating PDO formation. It has been documented [51] that, as hosts for the production of chemicals and fuels, *Clostridium* organisms have the major advantage of using a broad spectrum of substrates deriving from biomass (hexoses, pentoses, oligosaccharides, xylans), and although not extensively studied, *Cpa* does not appear to be an exception to this assessment. Its genome codes the necessary genes that would make it an effective user of such a broad spectrum of substrates, on top of its powerful ability to effectively and quickly utilize crude glycerol, alone and in combination with other carbohydrates.

## Methods

### Bacterial growth and maintenance conditions

Strains used in this study are listed in Table 3. *C. pasteurianum* ATCC 6013 was grown anaerobically at 37 °C in liquid CGM (per liter of distilled water: KH_2_PO_4_, 0.75 g; K_2_HPO_4_, 0.75 g; MgSO_4_ [anhydrous], 0.348 g; MnSO_4_·H_2_0, 0.01 g; FeSO4·7H_2_0, 0.01 g; NaCl, 1.0 g; asparagine, 2.0 g; yeast extract, 5.0 g; sodium acetate, 2.46 g; (NH_4_)2SO_4_, 2.0 g; and para-aminobenzoic acid [PABA], 0.04 g; titrated to pH 6.8) with, unless otherwise stated, 80 g/L glucose as the main carbon source. *C. pasteurianum* ATCC 6013 was grown anaerobically at 37 °C on solid 2xYTG (pH 6.5) agar plates [52] supplemented with the appropriate antibiotic (erythromycin at 40 µg/ml for solid-medium plates and 100 µg/ml for liquid medium or thiamphenicol at 10 µg/ml). *C. pasteurianum* strains were stored at −85 °C in CGM supplemented with 15 % glycerol and were revived by plating onto 2xYTG (pH 6.5) agar plates. *Escherichia coli* strains were grown aerobically at 37 °C in liquid LB medium or on solid LB agar plates supplemented with the appropriate antibiotic (50 µg/mL ampicillin, 35 µg/mL chloramphenicol, 25 µg/mL kanamycin). *E. coli* strains were stored at −85 °C in LB medium supplemented with 15 % glycerol.

**Table 3:** Strains and plasmids used in this study

	Description	Source
Strain		
*Cpa* ATCC 6013	Wild-type *Clostridium pasteurianum*	ATCC
*Cpa* M150B	Mutant isolated from crude glycerol selection	This study
* Cpa* ΔSpo0A	Constructed Spo0A deficient strain	This study
NEB Turbo	Cloning strain for DNA manipulation	NEB
Plasmid		
pCpaDcm2.0	Methylating plasmid	DNA 2.0
pKO-mazF	General recombination plasmid	Al-Hinai et al. [41]
pKO-mazF-spo0A	Knockout plasmid targeting Spo0A	This study
p94-Flp	Flippase plasmid for ThR removal	Al-Hinai et al. [41]

### Analytical methods

Cell density was measured at 600 nm using a Beckman Coulter DU 730 spectrophotometer. Samples were diluted with the appropriate medium to maintain readings in the linear range (below 0.40). DNA concentrations and purities were measured at 260 and 280 nm using a NanoDrop (Wilmington, DE, USA) spectrophotometer.

Culture supernatants were analyzed for acetate, butyrate, acetone, butanol, ethanol, acetoin, 1,3-propanediol, and glycerol concentrations using an Agilent LC high-performance liquid chromatography system (with 1260 Infinity standard autosampler, isocratic pump, refractive index detector, and in-line vacuum degasser) and Agilent ChemStation software. A Bio-Rad Aminex HPX-87H anion exchange column was used with a mobile phase of 0.05 mM sulfuric acid flowing at 0.50 mL/min. No acetoin was detected in any cultures. Lactic acid concentration in the supernatant was determined by a YSI 2700 biochemistry analyzer (YSI, Yellow Springs, OH, USA).

### Growth and metabolite assays

*Cpa* overnight CGM cultures were inoculated with individual colonies taken from solid media. For glycerol studies, a starter culture of CGM supplemented with 60 g/L molecular biology grade glycerol (no glucose) was inoculated with overnight culture to an OD = 0.020–0.050 and allowed to grow to mid-exponential phase (OD 0.4–0.8). Experimental cultures of 20–80 mL CGM supplemented with 60 g/L molecular biology grade glycerol or 100 g/L crude glycerol were inoculated from starter cultures to an OD = 0.070. All media was stored at 37 °C anaerobically for at least 48 h prior to use. Samples were taken for turbidity and metabolite analysis simultaneously. For metabolite analysis, samples were centrifuged for 1 min at 16,000 rcf, and the supernatant was removed and stored at −20 °C until analyzed. Samples were filtered with a 0.22 µm syringe filter prior to downstream analysis.

### Whole-genome SMRT sequencing

High molecular weight genomic DNA was isolated from *C. pasteurianum* ATCC 6013 and M150B strains using Genomic-tip 100/G (QIAGEN) according the manufacturer’s instructions. Single molecule real time sequencing (SMRT) sequencing was performed at the University of Delaware DNA Sequencing and Genotyping Center. SMRTbell DNA libraries were constructed according to the Pacbio standard protocol. Template preparation was performed using BluePippin (Sage Science) size-selection system including DNA damage and end repair steps and ligation to hairpin adapters. Both libraries were size-selected starting at 7 kb and with an average library size of 20 kb as measured by Fragment Analyzer (Advanced Analytical Technologies, Inc). Sequencing was performed on PacBio RSII (Pacific Biosciences, Menlo Park, California) instrument using P4-C2 chemistry, mag-bead loading and 3-h movie time. To identify consensus and variant sequences, quality-filtered reads were mapped against a reference sequence (accession number CP009267 [20]) using the RS Resequencing protocol within the SMRT Analysis version 2.3 through the SMRT Portal. The RS Modification and Motif Analysis protocol was used to identify base modifications and methyltransferase recognition motifs.

### Knockout of *Cpaspo0A*

Plasmids used in this study are listed in Table 3. Oligonucleotides were synthesized by Integrated DNA Technologies (IDT, Iowa City, IA, USA) and are listed in Additional file 1: Table S2. Two ~500 bp regions of homology designed to surround the ribosomal-binding site and first section of the *spo0A* gene (including the start codon) were amplified with primers 140 & 141 and 142 & 143. These were sequentially cloned into the pKO-mazF vector [41] using SphI & AgeI and MluI & NcoI to generate pKO-mazF-spo0A. Successful cloning at each stage was confirmed by PCR and Sanger sequencing.

Newly constructed plasmids were transformed into *E. coli* Turbo (NEB) cells and subsequently isolated and confirmed via PCR confirmation and Sanger sequencing. Immediately prior to transformation of plasmids into *Cpa*, plasmids were electroporated with *E. coli* ER1821(pCpaDcm2.0) (NEB). For plasmid methylation, cultures were supplemented with 1 mM rhamnose at least 4 h prior to plasmid extraction via miniprep. At least 500 ng of methylated plasmids were subsequently transformed into *Cpa* as described by Pyne et al. [11].

The *bepIM* gene was synthetically constructed (DNA 2.0, Menlo Park, CA, USA) for optimized codon usage in *E. coli* (see Additional file 1: Table S1). The *bepIM* gene was cloned into plasmid pD881 (pCpaDcm2.0), which uses a rhamnose inducible promoter.

### Microscopy

For light microscopy, samples taken from 6 day old CGM culture were pelleted and washed twice with 1 % w/v NaCl. Slides were imaged using a Leica widefield microscope with phase contrast to distinguish cell morphology.

For transmission electron microscopy, *Cpa* strains cultured for 5 days in CGM were pelleted and the supernatant was removed. The bacterial pellet was treated with 16 % paraformaldehyde and 8 % glutaraldehyde to the culture medium for a final concentration of 2 % paraformaldehyde and 2 % glutaraldehyde in 0.1 M sodium cacodylate buffer and inverted multiple times to mix. Cultures were fixed for 2 h at room temperature, pelleted and resuspended in buffer. The cells were then pelleted and embedded with 4 % low melting point agarose and cut into 1–2 mm^3^ cubes. Samples were washed for 15 min thrice in 0.1 M sodium cacodylate buffer (pH 7.4) before fixation for 2 h in 1 % osmium tetroxide solution. Samples were then dehydrated in increasing concentrations of acetone washes for 15 min each (25, 50, 75, 95, 100, 100 %). The samples were infiltrated with 50/50 mixture of acetone/n-BGE for 30 min, then infiltrated with 100 % n-BGE for an additional 30 min. Samples were infiltrated with Quetol resin in increasing concentrations with n-BGE as diluent: 25 % overnight, 50 % for 8 h, 75 % overnight, 100 % for 8 h, 100 % overnight, and 100 % for 8 h. Samples were embedded in BEEM capsules with fresh resin and polymerized for 48 h at 60 °C. Blocks were sectioned and imaged as described previously [42]. All microscopy imaging was performed at Delaware Biotechnology Institute BioImaging Center.

## Additional file

10.1186/s13068-015-0408-7 **Table S1:** Nucleotide sequence variants from published data. **Table S2:** Sequence and features of pDcm2.0 with codon optimized *Cpa bepIM*. **Figure S1:** Phase-contrast microscopy of WT and M150B after 6 days. Phase-bright forespores observed in the WT and the asporogenous phenotype is apparent in the M150B strain. **Figure S2:** Whole genome SMRT sequencing coverage depth overage across reference. *Cpa* wild type ATCC 6013 and *Cpa* mutant M150B coverage across the reference genome. **Figure S3:** Cultures of the wild type, M150B, and ΔSpo0A Cpa strains after 5 days culturing. Cultures were allowed to rest without agitation. M150B cultures consistently settled quickly compared to wild type, while the ΔSpo0A consistently remained in suspension longer than the wild type. This shows the M150B phenotype is not due to the Spo0A deficiency.

## Abbreviations

*Cpa*: *Clostridium pasteurianum*
PDO: 1,3-propanediol
SMRT: single molecule real-time
WT: wild type
NTG: *N*-methyl-*N*′-nitro-*N*-nitrosoguanidine
CGM: clostridial growth media
*Cac*: *C. acetobutylicum*
*Cbe*: *C. beijerinckii*
IPD: interpulse duration ratio
MAMA: modification and motif analysis
