# Supplementary material for: Whole-genome sequence of an evolved Clostridium pasteurianum strain reveals Spo0A deficiency responsible for increased butanol production and superior growth
Source: Biotechnol Biofuels. 2015 Dec 24;8:227. doi: 10.1186/s13068-015-0408-7 (PMC4690370; doi:10.1186/s13068-015-0408-7)
Supplement: Supplementary file 1 — 10.1186/s13068-015-0408-7 Table S1: Nucleotide sequence variants from published data. Table S2: Sequence and features of pDcm2.0 with codon optimized Cpa bepIM. Figure S1: Phase-contrast microscopy of WT and M150B after 6 days. Phase-bright forespores observed in the WT and the asporogenous phenotype is apparent in the M150B strain. Figure S2: Whole genome SMRT sequencing coverage depth overage across reference. Cpa wild type ATCC 6013 and Cpa mutant M150B coverage across the reference genome. Figure S3: Cultures of the wild type, M150B, and ΔSpo0A Cpa strains after 5 days culturing. Cultures were allowed to rest without agitation. M150B cultures consistently settled quickly compared to wild type, while the ΔSpo0A consistently remained in suspension longer than the wild type. This shows the M150B phenotype is not due to the Spo0A deficiency. [file 13068_2015_408_MOESM1_ESM.docx]

Whole-genome sequence of an evolved *Clostridium pasteurianum* strain reveals Spo0A deficiency responsible for increased butanol production and superior growth

Nicholas R. Sandoval1, Keerthi P. Venkataramanan1, Theodore S. Groth1, and Eleftherios T. Papoutsakis1,2

1 Department of Chemical and Biomolecular Engineering & The Delaware Biotechnology Institute, University of Delaware, 15 Innovation Way, Newark, DE 19711, USA

2 Department of Biological Sciences, University of Delaware, USA

# **Supplemental Results**

## **SMRT sequencing coverage indicates regions of DNA modification**

The wild type gDNA was extracted during the transition phase while the M150B gDNA was extracted during midexponential phase. The wild type coverage appears to be even across the genome with two notable exceptions discussed below. The M150B coverage appears to be dependent on the distance from the origin of replication of the chromosome. As M150B was actively growing at the time of gDNA harvesting, we suppose the regions near the origin are more abundant at the beginning of DNA replication.

We observed a notable increase in the coverage around 3.40-3.51 MB for only the wild type strain. The region near 3.51 MB contains five transposase genes (c32470-c32510) which may affect coverage in this region.

We observed a sharp increase in coverage for both the wild type and mutant around 4.27-4.31 Mb (Supp. Fig. 2). This is possibly due to phage elements at this locus (*c40170*-*c40480*). This phenomenon of increased coverage during SMRT sequencing around probable phage genes has been observed recently in this research group in other *Clostridium* species.

**Table S1 – Nucleotide sequence variants from published data**

| **Location** | **Type** | **Variation** | **Mutation** | **Note** |
| --- | --- | --- | --- | --- |
| 142286 | Del. | A to - | Frame shift | IS116/IS110/IS902 family transposase |
| 226301 | Del. | T to - | Frame shift | Methyl-accepting chemotaxis protein TlpB |
| 635237 | Del. | TT to - | Frame shift | IS801 transposase |
| 698471 | Del. | T to - | Frame shift | Integrase catalytic subunit |
| 699139 | Del. | A to - |  | No CDS |
| 2883135 | Del. | T to - | Frame shift | In transposase |
| 4138602 | Del. | T to - |  | No CDS |
| 1015245 | Ins. | - to T | Frame shift | Phage integrase family protein |
| 986198 | Sub. | C to G | A373P | IS1604transposase |
| 1247868 | Sub. | G to A | A319V | IS1604transposase |
| 2019787 | Sub. | G to A | E210K | Hypothetical protein AJA47948.1 |
| 2020013 | Sub. | C to G | T285S | Hypothetical protein AJA47948.1 |
| 2055741 | Sub. | A to G | K255E | Spo0A Sequence here is similar to most *Clostridium* Spo0A sequences, including Cpa DSM 625([21](#_ENREF_21)) |
| 2265810 | Sub. | T to C | E264G | Phosphate-binding protein PstS |
| 3203809 | Sub. | A to G |  | No CDS |
| 3205294 | Sub. | A to G | S54G | General secretion pathway protein A |
| 3525518 | Sub. | C to T |  | No CDS |
| All with >66x coverage, compared with Rotta et al. 2015([20](#_ENREF_20)) | | | | |

## **Table S2 - Sequence and features of pDcm2.0 with codon optimized *Cpa bepIM* (NS_dcm_Cpa)**

LOCUS 3378 bp circular

DEFINITION [pD881:144954]

ACCESSION DNA2.0 Id:

SOURCE Synthetic

FEATURES Location/Qualifiers

source 1..3378

/organism=synthetic

gene 151..1278

/gene="NS_dcm_Cpa"

/label="NS_dcm_Cpa"

misc_feature complement(2378..3136)

/product="Kanamycin-r"

/label="Kanamycin-r"

misc_feature 1..114

/product="P_rhaBAD"

/label="P_rhaBAD"

misc_feature 138..150

/product="strong RBS"

/label="strong RBS"

misc_feature 151..1278

/product="insert:"

/label="insert:"

misc_feature 1295..1385

/product="Term_PhageT7"

/label="Term_PhageT7"

misc_feature 1497..2323

/product="Ori_p15a"

/label="Ori_p15a"

BASE COUNT 848 T 816 G 893 A 821 C

1 CACCACAATT CAGCAAATTG TGAACATCAT CACGTTCATC TTTCCCTGGT

51 TGCCAATGGC CCATTTTCCT GTCAGTAACG AGAAGGTCGC GAATTCAGGC

101 GCTTTTTAGA CTGGTCGTAA TGAAATTCTT TTTAAGAAGG AGATATACAT

151 ATGGAGCAGC TCAGCATTTT CAATAAAGTC GACGATTTCC AAGAGCAGCA

M E Q L S I F N K V D D F Q E Q Q Frame 1

201 AACGGACGAT CGCGAACTGT CTATTGAAGA GATCAACAAG TTTATCAACG

T D D R E L S I E E I N K F I N E Frame 1

251 AGCACAAACT GACCGAGCGT ATCGATATCA TTAACACGGA GAATGCAAGC

H K L T E R I D I I N T E N A S Frame 1

301 AAACGTCGCT TTACCATTCT GAGCCTGTTT AGCGGTTGTG GTGGTCTGGA

K R R F T I L S L F S G C G G L D Frame 1

351 CTTGGGCTTC AAGGGCGGCT TCACCTATTT ACACGGTAAT TACGAACGTA

L G F K G G F T Y L H G N Y E R N Frame 1

401 ATAACTTCGA CATCATTTGG GCGAATGAAA TCAACAGCCA AGCTGTTGAA

N F D I I W A N E I N S Q A V E Frame 1

451 ACCTATCGCT CCTATTTCGG TAACCATATT GTGTGCGAGG ACATCAATAA

T Y R S Y F G N H I V C E D I N N Frame 1

501 CATTCGTGAT GATGAATTTC CTCAAGCGGA CATCATTATC GGTGGTTTTC

I R D D E F P Q A D I I I G G F P Frame 1

551 CGTGTCAGGA CTTCAGCCTG GCTGGTAAAA AGCAAGGCTT GAACGTTGAG

C Q D F S L A G K K Q G L N V E Frame 1

601 CGCGGTCGTC TGTATTTGCA GATGAAGCGT GCGATTGATG CGGTTAAGCC

R G R L Y L Q M K R A I D A V K P Frame 1

651 AGTCGCTTTC ATCGCCGAGA ATGTTCGTAA TCTGATGGTC ATGGGTAACG

V A F I A E N V R N L M V M G N G Frame 1

701 GCGTCGTACT GAAAACGATC ATCGACGACT TCAAGCAGAG CGGTTACAAT

V V L K T I I D D F K Q S G Y N Frame 1

751 GTGTACTTTC ACCTGTACAA TGCAGCGAAC TACGGTGTTC CGCAGAACCG

V Y F H L Y N A A N Y G V P Q N R Frame 1

801 TGAGCGTGTG ATCATTTATG GCATTCGTGA GGATCTGAAC AATATTCCGT

E R V I I Y G I R E D L N N I P F Frame 1

851 TCATTCCGCT GGAAACCCAT AGCCTGTATA ACTGGGTGAC TGCCTCTGAG

I P L E T H S L Y N W V T A S E Frame 1

901 GCGATCGATG ATCTGTGGGA CAAACTGGAT ACCAACATCC CGAACCACAG

A I D D L W D K L D T N I P N H S Frame 1

951 CAGACGCGAC TACTCGAAAG CGAAGTTTTA CGAAGGCAAG CGTACGCAGG

R R D Y S K A K F Y E G K R T Q G Frame 1

1001 GTAATATCCG TATTCAGAGC GACAAGGTTG CGCCGACCAT TCGCGCAGAA

N I R I Q S D K V A P T I R A E Frame 1

1051 CACCACGGTA ATATCGAAGG TCATTACCGC ACCTACGGCG ACGAGTCCGA

H H G N I E G H Y R T Y G D E S D Frame 1

1101 TCTGTCCAAT TGGCGTCGCC TGAGCGTGCG TGAGTGCGCA CGTATTCAAA

L S N W R R L S V R E C A R I Q T Frame 1

1151 CGTTTCCGGA TGACTTCATT TTTCAGAGCG CAGCCAGCAG CGCGTATAAA

F P D D F I F Q S A A S S A Y K Frame 1

1201 CAAGTCGGCA ACGCAGTTCC GCCGGTGCTG GCGTGGAATA TTGCCCGTGC

Q V G N A V P P V L A W N I A R A Frame 1

1251 GCTGTTCCTG AGCTTGATCC GCATCAAAGG TTAGAGCGGC CGCCACCGCT

L F L S L I R I K G * Frame 1

1301 GAGCAATAAC TAGCATAACC CCTTGGGGCC TCTAAACGGG TCTTGAGGGG

1351 TTTTTTGCTG AAAGGAGGAA CTATATCCGG GTAACGAATT CAAGCTTGAT

1401 ATCATTCAGG ACGAGCCTCA GACTCCAGCG TAACTGGACT GCAATCAACT

1451 CACTGGCTCA CCTTCACGGG TGGGCCTTTC TTCGGTAGAA GTCTTCTTAA

1501 TAAGATGATC TTCTTGAGAT CGTTTTGGTC TGCGCGTAAT CTCTTGCTCT

1551 GAAAACGAAA AAACCGCCTT GCAGGGCGGT TTTTCGAAGG TTCTCTGAGC

1601 TACCAACTCT TTGAACCGAG GTAACTGGCT TGGAGGAGCG CAGTCACCAA

1651 AACTTGTCCT TTCAGTTTAG CCTTAACCGG CGCATGACTT CAAGACTAAC

1701 TCCTCTAAAT CAATTACCAG TGGCTGCTGC CAGTGGTGCT TTTGCATGTC

1751 TTTCCGGGTT GGACTCAAGA CGATAGTTAC CGGATAAGGC GCAGCGGTCG

1801 GACTGAACGG GGGGTTCGTG CATACAGTCC AGCTTGGAGC GAACTGCCTA

1851 CCCGGAACTG AGTGTCAGGC GTGGAATGAG ACAAACGCGG CCATAACAGC

1901 GGAATGACAC CGGTAAACCG AAAGGCAGGA ACAGGAGAGC GCACGAGGGA

1951 GCCGCCAGGG GGAAACGCCT GGTATCTTTA TAGTCCTGTC GGGTTTCGCC

2001 ACCACTGATT TGAGCGTCAG ATTTCGTGAT GCTTGTCAGG GGGGCGGAGC

2051 CTATGGAAAA ACGGCTTTGC CGCGGCCCTC TCACTTCCCT GTTAAGTATC

2101 TTCCTGGCAT CTTCCAGGAA ATCTCCGCCC CGTTCGTAAG CCATTTCCGC

2151 TCGCCGCAGT CGAACGACCG AGCGTAGCGA GTCAGTGAGC GAGGAAGCGG

2201 AATATATCCT GTATCACATA TTCTGCTGAC GCACCGGTGC AGCCTTTTTT

2251 CTCCTGCCAC ATGAAGCACT TCACTGACAC CCTCATCAGT GCCAACATAG

2301 TAAGCCAGTA TACACTCCGC TAGCGCAGAA AGGCCCACCC GAAGGTGAGC

2351 CAGGTGATTA CATTTGGGCC CTCATTAGAA AAACTCATCG AGCATCAAAT

2401 GAAATTGCAA TTTATTCATA TCAGGATTAT CAATACCATA TTTTTGAAAA

2451 AGCCGTTTCT GTAATGAAGG AGAAAACTCA CCGAGGCAGT TCCATAGGAT

2501 GGCAAGATCC TGGTATCGGT CTGCGATTCC GACTCGTCCA ACATCAATAC

2551 AACCTATTAA TTTCCCCTCG TCAAAAATAA GGTTATCAAG TGAGAAATCA

2601 CCATGAGTGA CGACTGAATC CGGTGAGAAT GGCAAAAGTT TATGCATTTC

2651 TTTCCAGACT TGTTCAACAG GCCAGCCATT ACGCTCGTCA TCAAAATCAC

2701 TCGCATCAAC CAAACCGTTA TTCATTCGTG ATTGCGCCTG AGCGAGGCGA

2751 AATACGCGAT CGCTGTTAAA AGGACAATTA CAAACAGGAA TCGAGTGCAA

2801 CCGGCGCAGG AACACTGCCA GCGCATCAAC AATATTTTCA CCTGAATCAG

2851 GATATTCTTC TAATACCTGG AACGCTGTTT TTCCGGGGAT CGCAGTGGTG

2901 AGTAACCATG CATCATCAGG AGTACGGATA AAATGCTTGA TGGTCGGAAG

2951 TGGCATAAAT TCCGTCAGCC AGTTTAGTCT GACCATCTCA TCTGTAACAT

3001 CATTGGCAAC GCTACCTTTG CCATGTTTCA GAAACAACTC TGGCGCATCG

3051 GGCTTCCCAT ACAAGCGATA GATTGTCGCA CCTGATTGCC CGACATTATC

3101 GCGAGCCCAT TTATACCCAT ATAAATCAGC ATCCATGTTG GAATTTAATC

3151 GCGGCCTCGA CGTTTCCCGT TGAATATGGC TCATAGCTCC TGAAAATCTC

3201 GATAACTCAA AAAATACGCC CGGTAGTGAT CTTATTTCAT TATGGTGAAA

3251 GTTGGAACCT CTTACGTGCC GATCAAGAAG ACGGTCAAAA GCCTCCGGTC

3301 GGAGGCTTTT GACTTTCTGC TATGGAGGTC AGGTATGATT TAAATGGTCA

3351 GTATTGAGCG ATATCTAGAG AATTCGTC

## **Table S3 – List of oligonucleotides used in this study**

| Primer | Sequence (5’-3’) | Description |
| --- | --- | --- |
| 129 | TGGGAATAGAAATATAAAGGGGAGT | Amplification and Sequencing of Cpa Spo0A |
| 130 | ACCCTAAAACTACTCTCAACCCA | Amplification and Sequencing of Cpa Spo0A |
| 140 | GAAATAGCATGCGGCGATGCACAGATACTTACAAC | *spo0A* homology region 1 For w/ SphI site |
| 141 | TCCATTACCGGTACTCCCCTTTATATTTCTATTCCCA | *spo0A* homology region 1 Rev w/ AgeI site |
| 142 | TTAGACACGCGTATGCCGCATTTGGATGGATTAG | *spo0A* homology region 2 For w/ MluI site |
| 143 | GTCTGACCATGGTATCGACTTGCCCTCTAGACCAG | *spo0A* homology region 2 Rev w/ NcoI site |

Supplementary Figures

*Wild Type Cpa 6013*

*Mutant M150B*

A

B


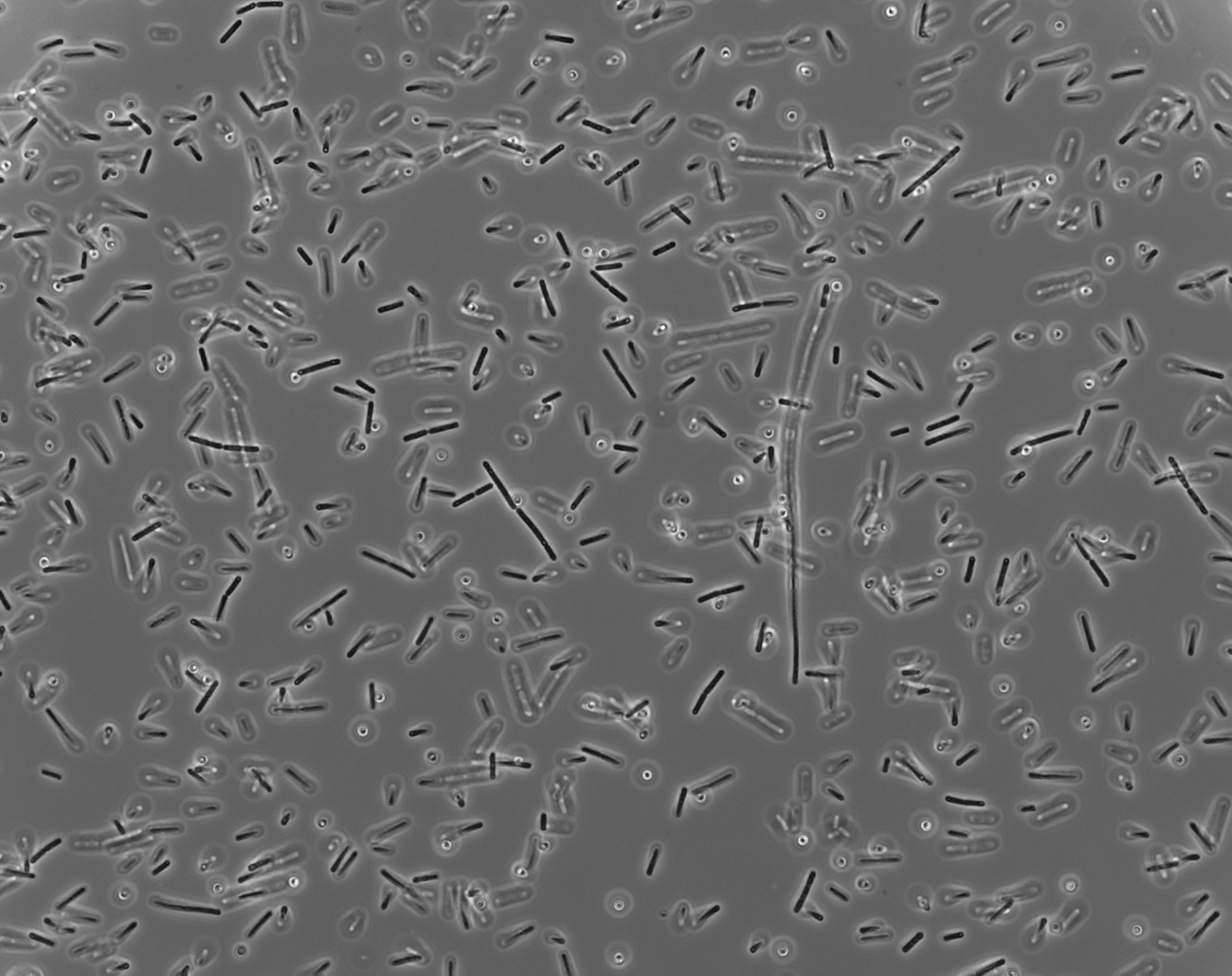

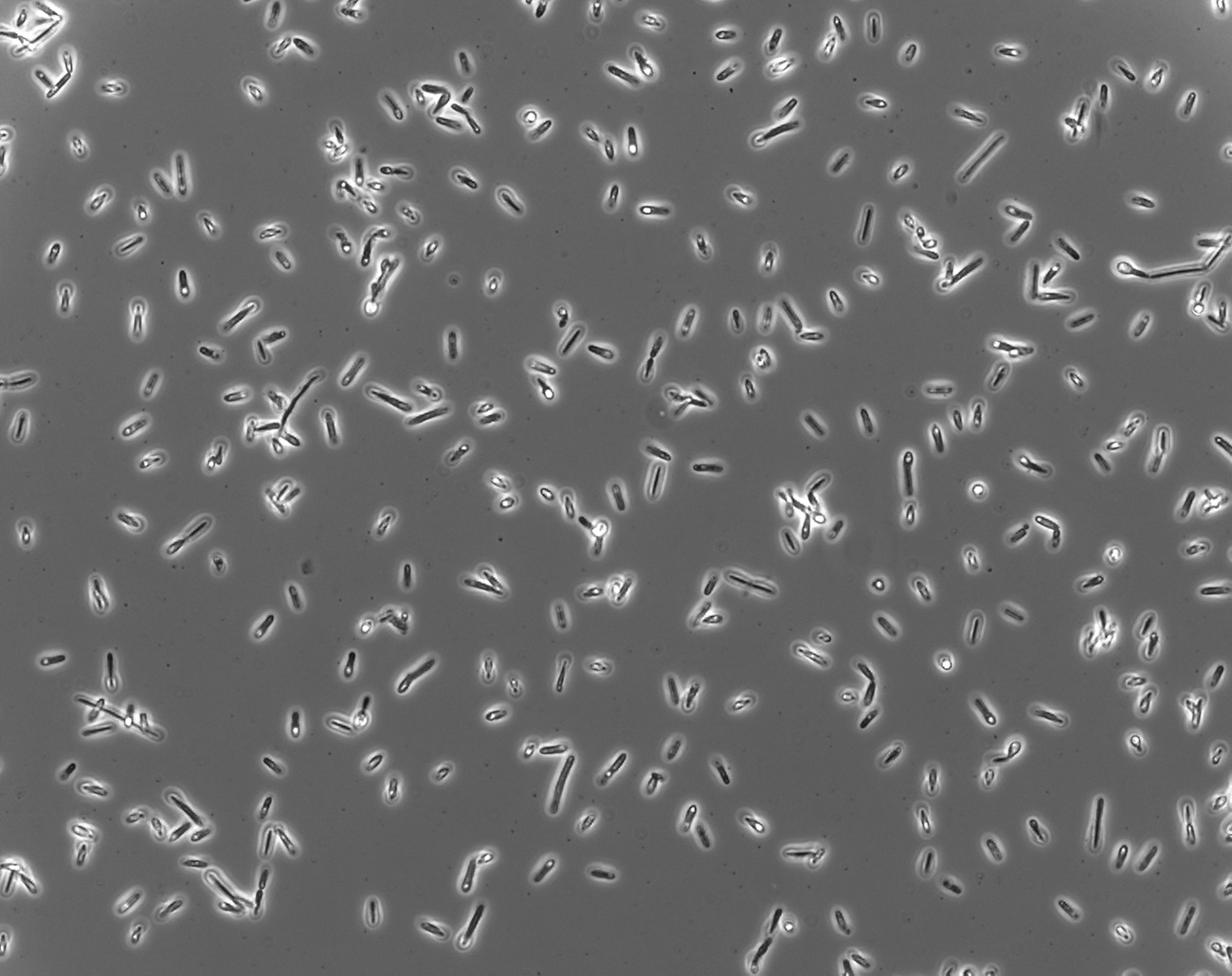


Figure S1. Phase-contrast microscopy of WT (A) and M150B (B) after 6 days. Phase-bright forespores observed in the WT and the asporogenous phenotype is apparent in the M150B strain.

Figure S2. Whole genome SMRT sequencing coverage depth overage across reference. *Cpa* wild type ATCC 6013 (olive) and *Cpa* mutant M150B (salmon) coverage across the reference genome (10,000 bp window average).


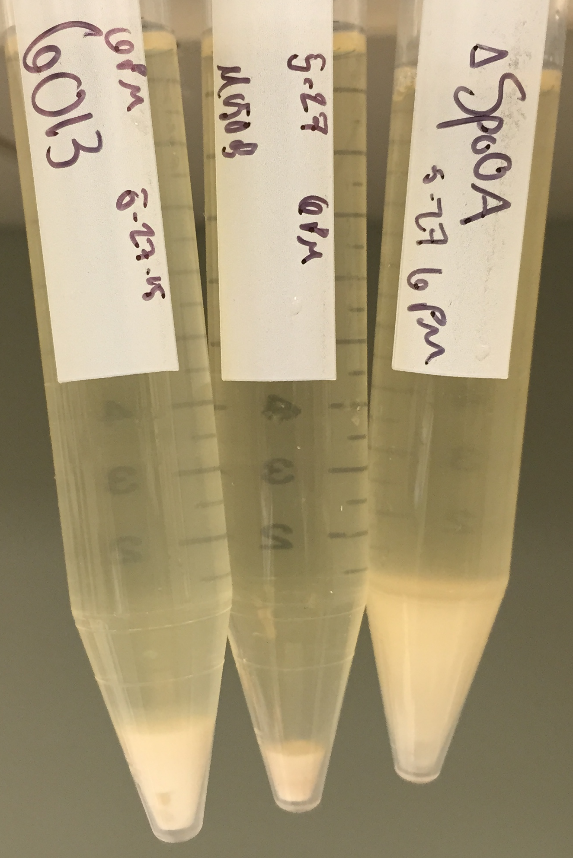


Figure S3. Cultures of the wild type (left), M150B (center), and ΔSpo0A (right) *Cpa* strains after 5 days culturing. Cultures were allowed to rest without agitation. M150B cultures consistently settled quickly compared to wild type, while the ΔSpo0A consistently remained in suspension longer than the wild type. This shows the M150B phenotype is not due to the Spo0A deficiency.
